# Preferences for mHealth Technology and Text Messaging Communication in Patients With Type 2 Diabetes: Qualitative Interview Study

**DOI:** 10.2196/25958

**Published:** 2021-06-11

**Authors:** Julie C Lauffenburger, Renee A Barlev, Ellen S Sears, Punam A Keller, Marie E McDonnell, Elad Yom-Tov, Constance P Fontanet, Kaitlin Hanken, Nancy Haff, Niteesh K Choudhry

**Affiliations:** 1 Brigham and Women's Hospital, Harvard Medical School Boston, MA United States; 2 Dartmouth College Dartmouth, NH United States; 3 Microsoft Research Herzliya Israel

**Keywords:** diabetes, technology, mobile health, medication adherence, mobile phone

## Abstract

**Background:**

Individuals with diabetes need regular support to help them manage their diabetes on their own, ideally delivered via mechanisms that they already use, such as their mobile phones. One reason for the modest effectiveness of prior technology-based interventions may be that the patient perspective has been insufficiently incorporated.

**Objective:**

This study aims to understand patients’ preferences for mobile health (mHealth) technology and how that technology can be integrated into patients’ routines, especially with regard to medication use.

**Methods:**

We conducted semistructured qualitative individual interviews with patients with type 2 diabetes from an urban health care system to elicit and explore their perspectives on diabetes medication–taking behaviors, daily patterns of using mobile technology, use of mHealth technology for diabetes care, acceptability of text messages to support medication adherence, and preferred framing of information within text messages to support diabetes care. The interviews were digitally recorded and transcribed. The data were analyzed using codes developed by the study team to generate themes, with representative quotations selected as illustrations.

**Results:**

We conducted interviews with 20 participants, of whom 12 (60%) were female and 9 (45%) were White; in addition, the participants’ mean glycated hemoglobin A_1c_ control was 7.8 (SD 1.1). Overall, 5 key themes were identified: patients try to incorporate *cues* into their routines to help them with consistent medication taking; many patients leverage some form of technology as a cue to support adherence to medication taking and diabetes self-management behaviors; patients value simplicity and integration of technology solutions used for diabetes care, managing medications, and communicating with health care providers; some patients express reluctance to rely on mobile technology for these diabetes care behaviors; and patients believe they prefer positively framed communication, but communication preferences are highly individualized.

**Conclusions:**

The participants expressed some hesitation about using mobile technology in supporting diabetes self-management but have largely incorporated it or are open to incorporating it as a cue to make medication taking more automatic and less burdensome. When using technology to support diabetes self-management, participants exhibited individualized preferences, but overall, they preferred simple and positively framed communication. mHealth interventions may be improved by focusing on integrating them easily into daily routines and increasing the customization of content.

## Introduction

### Background

Achieving optimal glycemic control substantially decreases the risk of long-term complications in individuals with type 2 diabetes [1-3]. Yet, more than 40% of patients with diabetes do not achieve their glycemic targets [4]. Medication adherence is an important factor in achieving diabetes control and improving clinical outcomes [5-8]. On average, less than half of patients take their medications as prescribed [7,8]. Managing diabetes also requires simultaneous adherence to diet and physical activity goals and achieving weight loss [5,9-12]. Although health care professionals can recommend treatments and promote healthy behaviors, activities like medication adherence, weight monitoring, dietary choices, and exercise must be self-managed by the patients [13,14]. Accordingly, to meaningfully improve outcomes, individuals with diabetes need regular support and feedback to help them manage these behaviors on their own, ideally delivered via mechanisms that they already use.

As of 2019, more than 80% of the US population owns smartphones, with relatively consistent uptake across most sociodemographic groups. Among the many functionalities of smartphones, text messaging is especially inexpensive and can provide reminders, education, and motivational support on an ongoing basis [15]. Growing evidence supports the effectiveness of text messaging–based approaches for diabetes self-management [15,16], although the magnitude of benefit that most patients receive is relatively modest [16-18].

One reason for this limited effectiveness of text messaging–based interventions in patients with diabetes may be that patients’ perspectives have been insufficiently incorporated into the message content and delivery. Little is known about specific preferences for mobile health (mHealth) technology for diabetes and how the technology could integrate into patients’ routines, especially with regard to medication use [19-21]. Patients may also exhibit specific preferences for how they receive text messaging–based communication from health care professionals, which may affect patients’ diabetes self-management activities.

### Objective

Therefore, we conducted in-depth, semistructured qualitative interviews with individuals with type 2 diabetes to elicit and explore their perspectives on (1) the medication-taking behaviors and challenges in diabetes; (2) their daily patterns of using mobile technology; (3) their use of mHealth technology for diabetes care, medication management, and provider communication; (4) the acceptability of a text messaging–based system for supporting medication adherence; and (5) their preferred framing of information within text messages to support diabetes care.

## Methods

### Study Participants and Recruitment

Participants were eligible if they were 18 years or older, diagnosed with type 2 diabetes, and were taking 1 or more prescribed oral medication for that condition. It should be noted that participants who were also using insulin were not explicitly excluded to enhance generalizability. We recruited a purposive sample of participants for qualitative interviews through direct clinician referral from Mass General Brigham health centers (formerly Partners Health care) as well as posting in and recruiting from a web-based database of participants who expressed an interest in participating in research studies in and around Boston, Massachusetts [22]. The recruitment and interviews occurred between January and March 2020. Informed consent was obtained from all interview participants, and they received a US $30 honorarium on the completion of the interview.

In this study, we followed the established standards for reporting qualitative research (ie, the COREQ [Consolidated Criteria for Reporting Qualitative Research] checklist) to ensure conformity with qualitative research standards, as recommended by the EQUATOR (Enhancing the Quality and Transparency of Health Research) Network [23,24]. Throughout the paper, we have referred to participants by their study ID to preserve anonymity. The Mass General Brigham Review Board approved this study.

### Qualitative Interviews

To elicit deeply personal accounts and develop a greater understanding of participants’ perceptions of diabetes management and the role of technology and text messaging–based solutions in their care, we chose to use semistructured, one-on-one qualitative interviews. To do so, the lead author, who had experience in qualitative methods, medication adherence, and clinical pharmacy, first drafted a comprehensive semistructured interview guide and circulated it for feedback and iterative refinement from other coinvestigators with expertise in qualitative methods, clinical medicine, mobile phone technology, diabetes, and medication adherence. Nonparticipant volunteers also pilot tested the guide, which led to no substantial changes. The guide covers separate but overlapping topics: (1) medication-taking behaviors and challenges in diabetes; (2) their use of mobile technology in their daily lives; (3) their use of technology for diabetes care, medication management, and provider communication; (4) general acceptability of a nonspecific text messaging–based system for medication management; and (5) preferred framing of information within text messages to support diabetes care (Multimedia Appendix 1, Table S1). The guide was used flexibly during the interviews to follow the natural flow of conversation and allow for free-flowing discussion by participants.

The participants were also shown 15 example text messages to respond to toward the end of the interview to further elicit perspectives on the framing of information. These text messages have been used in prior text messaging–based interventions for diabetes (Multimedia Appendix 1, Table S2) [25,26].

All interviews were conducted in English by a trained moderator who is also a practicing pharmacist (JCL). The interviews were conducted in person at secure locations around the Brigham and Women’s Hospital. At the beginning of the interview, the participants completed a baseline questionnaire that included questions about self-reported adherence to medication [27,28] and patient activation [29,30] (Multimedia Appendix 1, Figure S1). Each interview lasted between 30 and 75 minutes (mean 38.5, SD 10.3), and a sequential identification number was assigned to each interview. We conducted interviews until saturation was reached; saturation of the data was reached by patient 19 and reaffirmed with patient 20.

### Data Analyses

The audio-recorded interviews were transcribed verbatim. Transcripts were checked for accuracy against the recordings. To conduct the data analysis, we used immersion-crystallization methods [31,32]. In brief, this approach involves immersing in the data and then crystalizing salient themes during this process. Three investigators independently annotated a selection of transcripts and devised preliminary topic codes, with major categories consisting of technology, health, medication, routine, and texting. Each transcript was analyzed by at least two reviewers. Preliminary coding revealed themes around (1) having cues in medication regimen, (2) preferences for simplicity in communication, (3) concern about the applicability of communication, (4) reluctance to rely on technology, and (5) preferences for positively framed texts. After discussion and review with the coinvestigators, the preliminary codes were revised and agreed upon. We also identified broad themes at this stage.

Dedoose software version 8.3.10 (SocioCultural Research Consultants, LLC) was used for storage, handling, and analysis of the data set. We continued the immersion-crystallization approach until all the data were examined. Several early themes were clear: the role of habits in medication management, tendencies to use technology for managing diabetes but also being reluctant to rely on it, the perceived need for technology to seamlessly integrate into routines to be useful, and preferences for positively framed health communication. After coding, all the transcripts were then reread to identify any additional themes.

## Results

### Overview

We conducted interviews with 20 participants, and their key baseline sociodemographic and clinical characteristics are shown in Table 1. Among the participants, 60% (12/20) were female and 45% (9/20) were White; in addition, they had a mean glycated hemoglobin A_1c_ of 7.8 (SD 1.1). Although 65% (13/20) of the participants reported missing at least one day of medication in the last 30 days, the overall rates of adherence were modestly high (ie, a mean of 1.15 days missed, SD 1.1) [27,28].

In the interviews, participants reflected in detail about their medication-taking behaviors and routines; how they integrate mobile technology in their daily lives; their experiences with using mHealth technology to manage their diabetes; and their preferred method of receipt and framing of health-related communication, especially with regard to their medications.

On the basis of these interviews, we identified 5 key themes related to medication taking, technology, and preferences, which are summarized in Textbox 1. We present each of these themes in more detail in the following sections, with representative quotations by participants from the transcripts shown in italics. We also share the specific reactions to potential text messages shown to the participants.

**Table 1:** Participant characteristics.

Participant ID	Age (years)	Gender	Race and ethnicity	Education level	Medications in regimen, n	Latest glycated hemoglobin A_1c_ value	Days that medications were missed in the last 30 days, n
P1	57	Male	White	Some college	15	7.2	1
P2	36	Female	Black, Latino	Some college	3	9.2	2
P3	42	Female	White	Some college	3	6.4	0
P4	68	Male	White	College graduate	6	—^a^	1
P5	65	Male	Black	Some college	7	—^a^	2
P6	54	Female	Black	College graduate	4	9.1	0
P7	56	Male	White	Some college	10	9.7	2
P8	56	Female	Asian	Postgraduate	14	6.4	2
P9	55	Female	Black	College graduate	2	7.3	0
P10	48	Female	Black	Some college	14	6.9	3
P11	45	Female	White	College graduate	4	6.8	0
P12	54	Male	White	Postgraduate	9	7.1	1
P13	78	Male	White	College graduate	8	9.3	1
P14	63	Male	Black, native American	Postgraduate	7	7.3	1
P15	71	Male	White	Postgraduate	8	8.6	1
P16	56	Female	Black	Some college	5	8.0	0
P17	59	Female	Latino	Some college	3	7.1	0
P18	21	Female	Latino	Some college	1	7.0	3
P19	58	Female	Black	Some college	14	6.9	0
P20	47	Female	White	Postgraduate	3	8.0	3

^a^Data not reported.

**Summary of key themes.**

**Themes and key takeaways**

1. Patients try to incorporate cues into their routines to help them with consistent medication taking:
  Value of establishing a daily routine in supporting adherence
  Primarily use sight cues or habit cues
2. Many patients leverage some form of technology as a cue to support adherence to medication taking and diabetes self-management behaviors:
  Mobile phones serve as sight or habit cue for medication taking, either deliberately or accidentally
3. Value of simplicity and integration in technology solutions used for diabetes care, managing medications, and communicating with health care providers:
  Simplicity in communication and integration into routine can prevent exhaustion from being connected
  Straightforward, direct communication is preferred
4. Some patients express a reluctance to rely on mobile technology for these diabetes care behaviors:
  Concern about integration of mobile phones into daily lives for diabetes because of obsession with control values or concerns about screen time
5. Patients believe they prefer positively framed communication, but communication preferences are highly individualized:
  General preference for positively framed information
  Concerns about relevance of information to themselves or others, including advice and describing social support

### Patients Try to Incorporate Cues Into Their Routines to Help Them With Consistent Medication Taking

Participants described in great detail the value of a routine in supporting daily adherence to their diabetes medications, such as maintaining specific wake-up times, integrating medication taking into their bathroom use, or eating breakfast at the same time as their medications. Their medication-taking routines are most commonly supported by event-based cues centered on activities that are part of their normal routine, such as drinking coffee or using the bathroom or their mobile phone. These were sometimes supported by visual cues, such as sticky notes or a colorful pillbox in an obvious location. Others describe the act of glucose testing itself as supporting their medication taking and, therefore, the coupling of several activities that contribute to better blood sugar control. Conversely, for medications intended to be taken once a week, participants commonly described temporal cues, such as the day of the week:

If I remember church, I can remember to take the medication on Sundays.

P16

I like little hacks; trying to remember little hacks like by the coffee pot or maybe in the restroom in the morning. Where you’re brushing your teeth and you see your package of pills.

P4

Even if my day is different—like I’m out all day today, so I’ll probably have dinner before I go home, but as soon as I go home my pills are on my computer, which is one of the first things I do, so I take ‘em right away.

P1

My container is sitting right there [by the keys on the kitchen counter], so there’s a good chance I’m gonna look at it before I walk out the door. That’s the strategy.

P12

It’s automatic, almost. It’s almost part of getting my first cup of coffee. I put my machine in, press it to get the coffee, and then while it’s coming, I’ll go over and test.

P15

I’m programmed. I wake up. I make sure my coffee is brewing and then the pill before the coffee. That’s like a religion for me.

P17

Participants who largely reported a less *automatic* routine struggled with remembering to take their medications, filling their pillboxes, or recalling later whether they actually took their medications or not. Weekends appeared particularly difficult for participants because of the break in routine. Relatively few participants described behaviors that were fully automatic:

It’s not that I don’t remember the box. It’s that I don’t remember to fill certain medications in the box.

P8

The weekend is the hardest because in the weekend, routines change a little. I’m not rushing.

P17

Sometimes I forget, I get confused, and I don’t remember that I have already taken it or not. If I take another one, then I feel worse because my sugar levels are really low. I think that’s the hard part—to remember if I had it or not.

P18

I’m usually doing other things while I’m trying to remember the medication and get the kids ready. “Did I take the medication? I don’t remember.” Sometimes I didn’t even realize I missed one.

P20

### Many Patients Leverage Some Form of Technology as a Cue to Support Adherence to Medication Taking and Diabetes Self-management Behaviors

For about half of the participants, their mobile phones served as cues for medication taking. In some cases, participants generated specific cues using their phones as reminders, such as alarms or calendar reminders. In other cases, participants described physically storing their medications near their phone during the day because they looked at their phones frequently and therefore used them as direct cues. Others recognized that using their phones or other technology would be difficult to support medication taking if they were not seamlessly integrated into their daily lives. Whether participants deliberately used their phones for medication taking appeared to depend in part on how strongly they felt they needed that as a cue or how strongly they felt most generally about their need for cues for medication taking:

The eight AM one I have missed. I usually have it in my pocket for the most part. The 12:00 one I don’t miss ‘cause it’s usually in this pocket with the phone, and this phone comes in and out of my pocket all day long.

P1

I always set two [alarms] to take medication; usually if I took it, I uncheck the other one so the alarm doesn’t go off. If it does go off, that means I didn’t take it. I have to do it, otherwise there’s no way I’m gonna remember.

P2

P8 explained why a phone may not always work by stating the following:

It’s easier for men, because they have more pockets. I feel like I don’t [notice it]; I have this cross-body thing [bag] that can hold my phone.

P12 explained why he uses his phone’s calendar feature:

Because they remind me, and it’s something I don’t have to store in my head. It’s just that some things are better left to a machine and automation.

P14 stated the following:

The cell phone could be a screwdriver, or a hammer. It’s a tool. I don’t have an emotional attachment to it. Now I’m gonna sound like a boomer. I got along without ‘em. To me, it’s just a tool, not the center of my life.

### Patients Value Simplicity and Integration for Technology Solutions Used for Diabetes Care, Managing Medications, and Communicating With Health Care Providers

During the interview, participants reiterated that they disliked complicated technology or repetitive communication and offered several personal examples about likes and dislikes of communication that they currently receive. Simplicity was also valued because of its benefits in preventing exhaustion that comes from being constantly connected to technology and using technology for diabetes care. About one-third of the participants stated that they preferred written mobile communication rather than telephone calls, in part because of this desire for straightforwardness:

I just want you to get to the point and move on. Especially if you’re gonna remind me to take my pills. I mean it’s like did you take your pills today? You can either sit there and wait for a yes or a no. it’s very simple. Simplicity; I like simplicity.

P1

I love the little idea of—I love how straightforward the Brigham one [text] is. I love that. “Hey, did you take your meds? Respond back yes or no.” I think that’s great. Just straightforward.

P3

I hate talking on the phone, so if I could have done that on my phone, like in an app or texting, would’ve been 1000 times—‘cause I kept putting it off because I hate talking on the phone. Anything I don’t have to do face to face and can actually text or something, way better.

P11

Personally, I feel that watching out for what you have to eat and taking the pills and all this is frustrating in life. The longer the text message is the more like we’re like, “Blah.” We don’t pay attention to it ‘cause we don’t want more stress. We don’t want somebody telling, giving you all this lecture.

P17

In another context, when specifically asked about whether they would use text messages for diabetes, the concept was largely received positively, as long as the information delivered was simple and considered actionable. Participants largely discussed managing their diabetes as something within their locus of control, or their own responsibility, and noted that text messages would have to adapt to that. Specific feedback on each example text message shown to the participants is displayed in Multimedia Appendix 1, Table S2:

I think that would be short, to the point at the time when you should be takin’ it because texts, I think, are close to instantaneous to people. Most people have their phone in their pocket, especially the younger crowd. Older people, maybe not so much, but I think, even my generation is really into the phone, too. We mimic the younger kids because they do all the innovation and then show us how to do it.

P12

The daily stuff, I think I have it pretty much under control. I don’t think I need constant reminders, but I can see at some point it probably wouldn’t hurt.

P7

I like to text. I like to communicate. Communication is vital in my life and also with my own PCP [primary care physician], my own doctor, and all in your family and all. Yeah, so I like texts.

P17

### Some Patients Express Reluctance to Rely on Mobile Technology for These Diabetes Care Behaviors

Although participants largely reported high integration of mobile phones into their daily lives, they also expressed concern about this practice. In particular, they felt that technology could lead to an obsession with improving their diabetes numbers because of the rapid acquisition of those data. They described the physical exhaustion that comes from constantly concentrating on disease management, which may be amplified by technology use. There were also some stated concerns about overall screen time that led them to actively ignore or turn off some features that may be necessary to depend on mobile technology for diabetes care. Reluctance to rely on mobile technology may be associated with beliefs that technology is intrusive or patients are relinquishing control of their diabetes self-management:

You have to be your own advocate, but it’s a full-time job for me to manage my healthcare around my doctors and my prescriptions and, you know, everything. It’s just there are days where I just shut it off [the phone] and say, I can’t today. I try to limit screen time anyways; my phone will be at the bottom of my bag, and it might go off, and I just won’t hear it.

P8

It’s almost that I have a love hate relationship with the thing. I love it when I’m low. I hate it when I’m high. I tend to rely on my A_1C_ as my overall measure of how well I’m doing with my diabetes.

P12

My phone is always on silent. I can’t stand the noise.

P11

### Patients Believe They Prefer Positively Framed Communication, but Communication Preferences Are Highly Individualized

Participants expressed strong preferences for positively framed information within the communication they receive from health care providers. In their mind, positively framed communication involved motivational statements rather than penalizing statements or statements about the physical consequences of not taking medications. They also articulated similar desires for text messages to support medication use:

Because I’m into positive thinking in the way of trying to stay positive, trying to say things positive, so I like it when people send you those messages.

P5

More of encouragement than, “You missed this. You didn’t do this,” more of the whole, “Let’s get this done. Did you do”—more encouragement than penalized, feeling penalized.

P7

Trying to find something positive, either a way to do something positive or a positive outcome of, “If you do this—”

P11

Overall, participants wanted customized communication, and unless the information was applicable to them (eg, if they did not have family members involved in their care or social support), they surmised that they would likely ignore the text. Participants did not like texts that they deemed unrelated to their personal routine, such as texts with tips or advice. When asked about other types of communication preferences, such as whether to provide additional information beyond reminders, such as specific links to more information about diabetes self-care, or mentioning friends or family as sources of social support, participants had varied opinions about whether that would help them support medication taking:

People do things different ways. I was getting a text from somebody, I would wanna make it feel personal because then you tend not to ignore it than if it’s a generic.

P9

Friends and family’s good, but friends and family a lotta times tend to be on the negative side of things and not as encouraging as you’d like, more, “You didn’t do it, so you’re bad.”

P7

To point out that friends or family could help me—but it’s, like, “Well, they’re not around.”

P3

I can’t tell my family [about my diabetes] ‘cause they’re very judge-y. I can’t stand when people micro-watch you. I don’t feel comfortable telling by family because they’ll be, like, “Well, can you eat that? Can you do that?”

P11

It’s pretty much known that if you eat well and exercise 30 minutes a day. I don’t need to be reminded of that.

P6

## Discussion

### Principal Findings

There is an urgent need to identify effective and sustainable strategies to engage individuals with type 2 diabetes on an ongoing basis and in their usual environments. mHealth technology has the potential to support self-care activities, only if its delivery of communication could be optimized [33]. In this qualitative interview study, participants expressed some hesitation about the role of mobile technology in supporting diabetes self-management but have largely incorporated or are open to incorporating it as a cue in the pursuit of making medication taking more automatic and less burdensome. When using technology to support diabetes self-management, participants generally preferred simple and positively framed communication.

### Comparison With Prior Work

Of the few prior studies evaluating preferences for mHealth technology in diabetes, one conducted among 15 participants in the United Kingdom found a similar *love-hate* relationship with the increased awareness of glucose control provided by mHealth [34]. In another study of focus groups with 23 participants in the United Kingdom that concentrated on the acceptability of text messaging, participants relayed similar concerns about the relevance of content and expressed similar reluctance to rely on technology, largely because they felt that they should take personal responsibility for remembering to take medications [20]. Our study builds on this existing research but provides further insight into the role of mHealth in medication taking and preferences regarding message framing. Although preferences for simplicity have been observed in prior studies [35], it is notable that our US participants emphasized its importance in the context of reducing cognitive load and screen time [20,34,36,37].

Our findings also differ somewhat from other prior qualitative studies of text messaging, as our participants placed less emphasis on texts that invoke social concepts. A qualitative interview study in Argentina found that patients appreciated socially framed content in text messages [38], a finding consistent with survey data showing modestly high preferences for texts incorporating social support [21]. These differences could be attributable to culture or preferences for social support across different countries and populations, possibly because culture in the United States is generally more individualistic [39-42].

Related literature beyond text messaging programs about mobile apps reveals findings that are similar to ours. Patients have strong preferences for diabetes apps that are easy and efficient to use [43,44], and strong integration in patients’ daily routines has also been noted as desired features of mobile apps [45,46]. Similarly, communication preferences themselves have been thought to be highly individual [47]. However, several differences between mobile apps and text messaging programs should be noted: (1) participants must have a smartphone to use a mobile app, (2) participants must more actively open or engage with apps to derive benefits, and (3) participants express specific financial concerns about apps [44,46]. These considerations may be less relevant to mHealth messaging programs [15,44].

### Implications of the Findings

These findings offer several lessons for improving mHealth messaging programs for diabetes. Participants identified several strategies that are consistent with evidence from behavioral science, such as salience and framing of information [48-50]. For example, one key concept from the interviews was the desire for applicability and customization to ensure that the content is relevant and, therefore, salient to patients. Other limited existing evidence also suggests that mobile technology–based interventions may be most effective when information is tailored to the characteristics of individual patients, such as their specific barriers to adherence; of course, not all barriers can be easily addressed in text message solutions [51,52]. Furthermore, participants identified the importance of framing of communication, which is of demonstrated importance in other contexts, such as preventive health screenings and vaccinations [48,49,53]. These interviews suggested that framing, especially positively, may affect influence whether patients respond to text messaging programs in diabetes. Although, on average, patients prefer positive framing, persuasiveness of other framing may depend on patients’ specific barriers, and framing in text messaging programs specifically may warrant further empirical exploration.

Other design factors also appear to be important for the success of text messaging–based interventions in diabetes. The simplicity and ease of incorporating a hypothetical text messaging–based system into patients’ daily lives appear to be central to engendering the automaticity in medication taking that patients aim for. Mobile phones may already serve as habit-based cues, which could support patients until they no longer need to rely on those cues. However, there may be other ways in which text messaging programs should be better integrated into clinical care, such as more seamless communication with patient portals or electronic health record systems. Overall, technology can overcome many medication adherence barriers, including enhancing planning, being objective or not judgmental, and a ubiquitous presence, but more research is needed on the characteristics of patients who may benefit the most. Similarly, text messages for medication adherence could also be more persuasive than text messages for diet and exercise reminders because adherence behaviors are generally easier to implement; however, any differences are not yet well characterized.

### Limitations

This study has several limitations. First, this study was conducted in an urban academic medical center in eastern Massachusetts and recruited directly from clinics or from a web-based database of interested subjects, which could have affected generalizability; however, we enrolled a clinically and demographically diverse sample of participants. Second, the mean age of the study sample was 54 years (SD 12.3), which reflects the age distributions of individuals with type 2 diabetes in the United States; nevertheless, the results could have underrepresented younger viewpoints, who may have differing perspectives on technology use [54]. Third, because we conducted the interviews in person, response bias may have been possible. Finally, although participants using insulin were not excluded, the results may not be generalizable to patients exclusively using injectables to manage their diabetes.

### Conclusions

Participants appeared to express some trepidation about the daily role of mobile technology, but they have largely incorporated it or are open to incorporating it as a cue in the pursuit of making medication taking more automatic and less burdensome. mHealth interventions may be improved by focusing on easy integration into daily routines and increasing personalization. Careful, tailored application of behavioral science theories may be especially important in a society that increasingly relies on at-home, virtual care for managing diabetes.

## Appendix

Multimedia Appendix 1 Supplementary tables and figure.

## Abbreviations

COREQ: Consolidated Criteria for Reporting Qualitative Research
EQUATOR: Enhancing the Quality and Transparency of Health Research
mHealth: mobile health
