# Supplementary material for: Preferences for mHealth Technology and Text Messaging Communication in Patients With Type 2 Diabetes: Qualitative Interview Study
Source: J Med Internet Res. 2021 Jun 11;23(6):e25958. doi: 10.2196/25958 (PMC8235286; doi:10.2196/25958)
Supplement: Multimedia Appendix 1 [file jmir_v23i6e25958_app1.docx]

**SUPPLEMENTARY APPENDIX**

**Appendix Table 1.** Semi-structured interview guide

| **Overall topics** | **Types of questions** |
| --- | --- |
| **How patients are using their medications and medication-taking challenges** | - Walk me through a typical day in taking medication. What does your routine look like? - What challenges do you most experience in taking your medications regularly? If you’ve had any problems, how do you solve them? |
| **Daily patterns of using mobile technology** | - How often do you use your phone? - How many texts do you receive a day right now? What makes you respond differently to different types of messages? |
| **Use of technology for diabetes care, medications, and provider communication** | - What do you use to manage your diabetes? Do you use anything on your phone? - What types of apps or programs have you tried before? What have you tried for your medications? What do you wish you had available to you that you do not? - What type of communication do you receive from your doctor’s offices or pharmacies? What do you think about those messages? |
| **Acceptability of text messages to support adherence** | - How would you incorporate text messaging programs for your diabetes? - Would you need a text messaging program to help you with your diabetes? - What kind of texts do you think would be helpful to you to manage your medications? |
| **Framing of information within text messages** | - What type of message would you like to receive? - What do you think an optimal message would be for you about diabetes? - What type of message do you think would motivate you the most? - What do you think about these text messages (present example text messages)? |

**Appendix Table 2.** Example text messages and participant feedback to the messages

| **Example text message** | **Summary of participant feedback** |
| --- | --- |
| You have so far taken your medication all 7 days this week. Keep up the good work! | Mostly positive feedback due to encouraging, positive tone |
| You need to take your medications every day to prevent negative consequences. Please remember to take your medications. | Mixed feedback; some participants appreciated the reminder of negative consequences, others did not |
| Friends and family can help remind you to take your medications and be a good source of support. | Mostly negative feedback; many concerns about others not having friends and family to support them |
| Following a healthy lifestyle is the key to long-term health. The American Diabetes Association has some great tips at https://bit.ly/1eyJXE2 | Mostly positive feedback; some participants liked having information, others thought the information may not be necessary |
| Managing your health can be difficult. But exercising regularly can help prevent complications down the road. Stick to it! | Mostly positive feedback, due to motivational tone |
| Have you tried setting an alarm to help you take your medication? Try using your phone’s alarm or a tracker app on your smart phone. | Mostly positive feedback, except for those who believed they did not need to make modifications to their routine |
| Do you drink coffee in the morning? Try to put your medication by your coffee pot to help you remember to take your medication. | Mostly positive feedback, except for those who believed they would ignore the information when not applicable to them |
| When you are feeling well, do you sometimes stop taking your medications? Skipping medications may affect your long-term health. | Mixed feedback; some participants appreciated the reminder of negative consequences, others did not |
| Set short term goals. Try to take your medications every day for the next 7 days. Remember, missing doses is missing out on better health. | Mostly positive feedback due to the idea of goal-setting and motivational tone |
| In the last 7 days, how many days did you take all of your medications? Please text back the number of days you took your medications (0-7). | Mixed feedback; many liked that it was interactive, but others thought it could be annoying to have to respond |
| Ask a loved one to help you with your medications if you haven’t already. Remember you’re taking your medications for you and those who care about you. | Mostly negative feedback; many concerns about others not having friends and family to support them |
| Let’s start with small steps to improve. Think about the last time you didn’t take your medications. What can you do to keep it from happening again? | Mixed feedback; concerns about the complexity of the text language and framing |
| Please remember to take your medication now. | Mainly positive feedback due to simplicity of text message |
| Have you taken your medication yet? Please take your medication as prescribed by your health care provider. | Mostly positive feedback; some participants felt it was unnecessary to name provider |
| Remember, your medicine will work best if you also make healthy changes to how you eat and if you are active for least 30 minutes every day. | Mostly positive feedback, but some did not want to be reminded of lifestyle modifications |

**Appendix Figure 1.** Baseline questionnaire (3 pages)

**
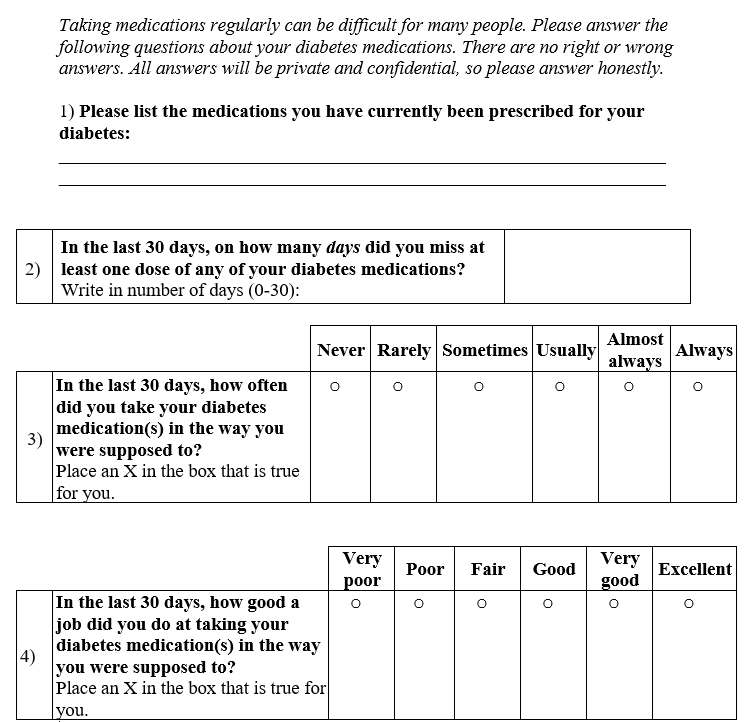
**

**
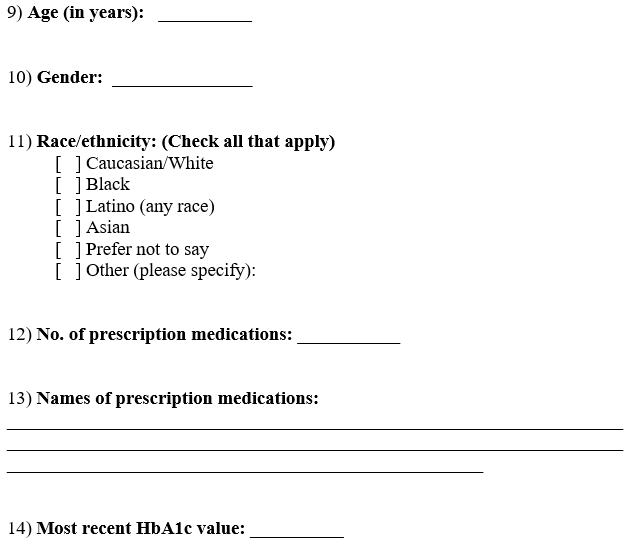

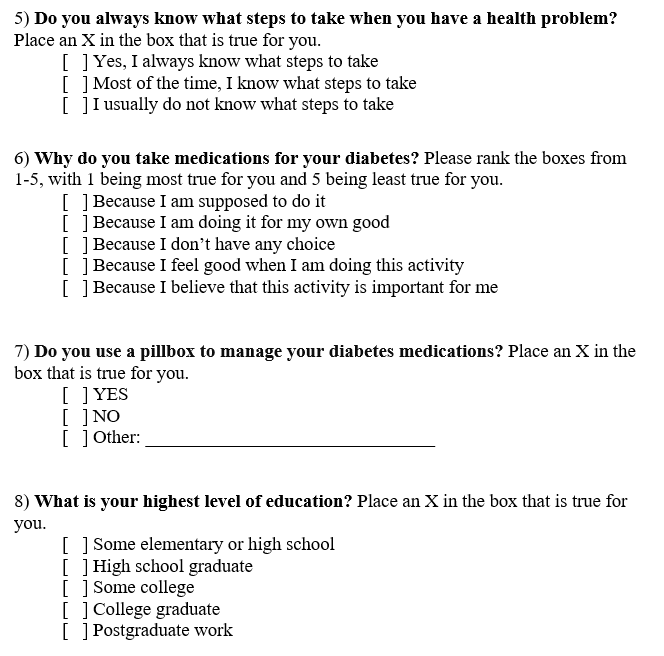
**
